# Biofilm formation and adherence characteristics of an *Elizabethkingia meningoseptica *isolate from *Oreochromis mossambicus*

**DOI:** 10.1186/1476-0711-10-16

**Published:** 2011-05-05

**Authors:** Anelet Jacobs, Hafizah Y Chenia

**Affiliations:** 1 Department of Microbiology, University of Stellenbosch, Private Bag X1, Matieland, 7602, South Africa; 2 Discipline: Microbiology, University of KwaZulu-Natal, Private Bag X54001, Durban, 4001, South Africa

**Keywords:** *Elizabethkingia meningoseptica*, tilapia, biofilm, adherence, autoaggregation, coaggregation

## Abstract

**Background:**

*Elizabethkingia *spp. are opportunistic pathogens often found associated with intravascular device-related bacteraemias and ventilator-associated pneumonia. Their ability to exist as biofilm structures has been alluded to but not extensively investigated.

**Methods:**

The ability of *Elizabethkingia meningoseptica *isolate CH2B from freshwater tilapia (*Oreochromis mossambicus*) and *E. meningoseptica *strain NCTC 10016^T ^to adhere to abiotic surfaces was investigated using microtiter plate adherence assays following exposure to varying physico-chemical challenges. The role of cell-surface properties was investigated using hydrophobicity (bacterial adherence to hydrocarbons), autoaggregation and coaggregation assays. The role of extracellular components in adherence was determined using reversal or inhibition of coaggregation assays in conjunction with *Listeria *spp. isolates, while the role of cell-free supernatants, from diverse bacteria, in inducing enhanced adherence was investigated using microtitre plate assays. Biofilm architecture of isolate CH2B alone as well as in co-culture with *Listeria monocytogenes *was investigated using flow cells and microscopy.

**Results:**

*E. meningoseptica *isolates CH2B and NCTC 10016^T ^demonstrated stronger biofilm formation in nutrient-rich medium compared to nutrient-poor medium at both 21 and 37°C, respectively. Both isolates displayed a hydrophilic cell surface following the bacterial adherence to xylene assay. Varying autoaggregation and coaggregation indices were observed for the *E. meningoseptica *isolates. Coaggregation by isolate CH2B appeared to be strongest with foodborne pathogens like *Enterococcus, Staphylococcus *and *Listeria *spp. Partial inhibition of coaggregation was observed when isolate CH2B was treated with heat or protease exposure, suggesting the presence of heat-sensitive adhesins, although sugar treatment resulted in increased coaggregation and may be associated with a lactose-associated lectin or capsule-mediated attachment.

**Conclusions:**

*E. meningoseptica *isolate CH2B and strain NCTC 10016^T ^displayed a strong biofilm-forming phenotype which may play a role in its potential pathogenicity in both clinical and aquaculture environments. The ability of *E. meningoseptica *isolates to adhere to abiotic surfaces and form biofilm structures may result from the hydrophilic cell surface and multiple adhesins located around the cell.

## Background

Members of the genus *Elizabethkingia *are aerobic, non-motile, Gram-negative rods that display a light yellow pigmentation or may be non-pigmented [1]. The absence of gliding motility and the presence of flexirubin pigments differentiate these genera from other genera in their family Flavobacteriaceae. Only two species have been identified to date, i.e., *Elizabethkingia meningoseptica *and *E. miricola *[1].

*E. meningoseptica *is the most significant species for human clinical infections, although *E. miricola *has been associated with sepsis [2]. *Elizabethkingia*-related infections occur in severely immuno-compromised and post-operative patients as well as neonates [1]. *E. meningoseptica *has been implicated in endocarditis, cellulitis, abdominal infection, septic arthritis and eye infections in severely immuno-compromised patients [1] suffering from malignancy, end-stage hepatic and renal disease, extensive burns and acquired immune deficiency syndrome as well as community-acquired necrotizing fasciitis, pneumonia, and bacteraemia [3]. These infections constitute a major clinical concern, since together with *Chryseobacterium *spp., *Elizabethkingia *spp. isolates are constitutively resistant to multiple antibiotics [1,4].

*Elizabethkingia *spp, isolates constitute a further threat, being able to thrive in aqueous environments and are associated with intravascular device-related bacteraemias, wound sepsis, and ventilator-associated pneumonia by virtue of their ability to contaminate and persist in fluid-containing apparatus [2,3,5]. *E. meningoseptica *has been found in the hospital environment in such sites as water supplies, saline solution used for flashing procedures, disinfectants, and medical devices, including feeding tubes and arterial catheters [6]. Outbreaks have been documented following administration of contaminated medicine, use of devices contaminated via water or more sporadic infections in immuno-compromised patients or post-trauma and -surgery patients. The bacterium has been isolated from such medical devices as the respirator, vaporizer and artificial ventilation tubing. *E. meningoseptica *strains isolated from slimy biofilm communities inside spouts of sink taps of a hospital have been implicated in a neonatal meningitis outbreak [7].

*Elizabethkingia *spp. have also been isolated from diverse ecological niches, including eutrophic lakes, soil, freshwater sources, spent nuclear fuel pools, and water condensation on the Russian space laboratory Mir [1]. *E. meningoseptica *have been recovered from diverse eukaryotes, including amoebae, frogs, turtles, birds, cats, dogs, and fish. The first *E. meningoseptica *infection in fish was diagnosed in farmed koi carp with skin lesions and hemorrhagic septicaemia. Fish-associated members of the genus *Elizabethkingia *may represent pathogenic or spoilage organisms or belong to the normal bacterial flora that colonize the mucus at the surface of the skin and gills and the intestine of healthy fish [1].

In the aquaculture environment, two challenges may be posed by *E. meningoseptica*, i.e., ability of these multidrug-resistant species to evade eradication following antimicrobial treatment and persistence in tanks due to biofilm community formation, leading to disease and associated economic losses; and their potential role as opportunistic human pathogens. The ability of these organisms to act as potential zoonotic pathogens, via transmission from fish and fish farm environments to immuno-compromised workers and consumers should not be underestimated [4] and necessitates investigation into their ability to adhere to surfaces and form biofilms.

Although *Elizabethkingia *spp. isolates have been isolated from clinical biofilm communities [7,8], the factors involved in initiating biofilm formation by these non-motile bacteria has not been elucidated. The present study investigated the ability of *Elizabethkingia meningoseptica *isolate CH2B from farmed freshwater tilapia and strain NCTC 10016^T^, to adhere to polystyrene under various physico-chemical conditions using the microtiter plate assay. Hydrophobicity as well as coaggregation and autoaggregation abilities were also investigated. The role of extracellular cell components in adherence was determined using reversal and inhibition of coaggregation assays, while the effect of cell-free supernatants from diverse bacteria in inducing enhanced adherence was investigated using microtiter plate adherence assays. Biofilm architecture of *E. meningoseptica *isolate CH2B was examined using a flow cell system, as was the ability of *E. meningoseptica *isolate CH2B to form a mixed biofilm structure with *Listeria monocytogenes*.

## Methods

### Bacterial growth conditions and identification

Creamish-yellow pigmented isolate CH2B was cultured from diseased freshwater tilapia (*Oreochromis mossambicus*) from a South African aquaculture facility. Isolate CH2B was presumptively identified as *E. meningoseptica *using the following tests: Gram stain, colony characteristics, and flexirubin pigment production [1]; and this identification was confirmed by 16S rRNA gene PCR and sequencing [9] (GenBank: EU598807).

*E. meningoseptica *isolate CH2B and type strain NCTC 10016^T ^were maintained on enriched Anacker and Ordal's agar (EAOA) [10] at ambient temperature (21°C ± 2°C). For long-term storage, cultures were placed in 20% glycerol and enriched Anacker and Ordal's broth (EAOB) and stored at -80°C.

### Biofilm formation and quantification

*E. meningoseptica *isolates CH2B and NCTC 10016^T ^were cultured overnight in EAOB at room temperature (21°C ± 2°C) and centrifuged for 2 min at 12000 rpm. Cell pellets were washed and re-suspended in phosphate-buffered saline (PBS, pH 7.2) to a turbidity equivalent to a 0.5 M McFarland standard [11]. In order to determine bacterial microtitre plate adherence, wells of sterile 96-well U-bottomed polystyrene microtiter plates (Deltalabs S.L, Barcelona, Spain) were each filled with 90 μl EAOB/tryptic soy broth (TSB; Merck Chemicals, Gauteng, RSA) and inoculated with 10 μl of standardized cell suspensions, in triplicate [12]. Negative control wells containing only broth or PBS were included in each assay while a *Vibrio mimicus *isolate (VIB1; isolated from cultured trout) was used as a positive control. Plates were placed on a C1 platform shaker (New Brunswick Scientific, Edison, NJ, USA) and/or the benchtop to simulate dynamic and static conditions, respectively, and incubated aerobically at room temperature (21°C ± 2°C) and/or 37°C for 24 h, in either nutrient-poor EAOB/nutrient-rich TSB media. An optical density (OD) reading of each well was obtained at 595 nm using an automated microtiter-plate reader (Microplate Reader model 680, BioRad Laboratories Inc., Hercules, California). Tests were done in triplicate on three separate occasions and the results averaged [12].

Biofilm formation was classified as non-adherent, weakly-, moderately- or strongly-adherent. The cut-off OD (OD_c_) for the microtiter plate test was defined as three standard deviations above the mean OD of the negative control. Isolates were classified as follows: ODOD_C _= non-adherent, OD_C _< OD(2 × OD_C_) = weakly adherent; (2 × OD_C_) < OD ≤ (4 × OD_C_) = moderately adherent and (4 × OD_C_) < OD = strongly adherent [12]. Statistical significance of differences (*p *< 0.05) due to altered variables (temperature, medium, agitation) in the microtiter adherence assays were determined using one way repeated measures analysis of variance (ANOVA; SigmaStat, V3.5, Systat Software, Inc., USA).

### Bacterial adherence to hydrocarbon assay

Surface hydrophobicity was assessed using the bacterial adherence to hydrocarbons (BATH) assay, with xylene (BDH, VWR International, Leicestershire, UK) as the hydrocarbon of choice [11]. *E. meningoseptica *isolates CH2B and NCTC 10016^T ^grown in EAOB at room temperature (21°C ± 2°C) were harvested during the exponential growth phase (18 h old cultures), washed three times and resuspended in sterile 0.1 M phosphate buffer (pH 7) to an OD of 0.8 at a wavelength of 550 nm (A_0 _of 10^8 ^CFU/ml). Samples (3 ml) of the bacterial suspension were placed in glass tubes with 400 μl of xylene, equilibrated in a water bath at 25°C for 10 min and vortexed [11,13]. After a 15 min phase separation, the lower aqueous phase was removed and its OD_550 _determined (A_1_). Strains were considered strongly hydrophobic when values were >50%, moderately hydrophobic when values were in the range of 20-50%, and hydrophilic when values were <20% [14]. Each value represents the mean of experiments done in triplicate and on two separate occasions. PBS was used as a negative control and *V. mimicus *isolate VIB1 was used as a control [11].

For the modified salting aggregation test (SAT) assay, overnight EAOB cultures grown at room temperature (21°C ± 2°C) were harvested, washed twice and resuspended in PBS (pH 7.2). *E. meningoseptica *isolate CH2B was 'salted out' (aggregated) by combining 25 μl volumes, containing 2 × 10^9 ^bacteria, with 25 μl volumes of a series of methylene blue-containing ammonium sulphate [(NH_4_)_2_SO_4_] concentrations (0.2, 0.5, 1, 1.5, 2, 2.5, 3, and 4 M) on microscope slides [11]. The lowest final concentration of (NH_4_)_2_SO_4 _causing aggregation was recorded as the SAT value and classified as follows: < 0.1 M = highly hydrophobic, 0.1 M - 1.0 M = hydrophobic and >1.0 M = hydrophilic [15]. Experiments were done in triplicate on two separate occasions and respective (NH_4_)_2_SO_4 _concentrations were used as negative controls.

### Autoaggregation and coaggregation assays

For the autoaggregation assay, *E. meningoseptica *isolates CH2B and NCTC 10016^T ^were grown in 20 ml EAOB at room temperature (21°C ± 2°C), harvested after 36 h, washed and re-suspended in sterile distilled H_2_O to an OD of 0.3 at a wavelength of 660 nm. The percentage of autoaggregation was measured by transferring a 1 ml sample of bacterial suspension to a sterile plastic 2 ml cuvette and measuring the OD after 60 min using a DU 640 spectrophotometer (Beckman Coulter) at a wavelength of 660 nm [16]. The degree of autoaggregation was determined as the percent decrease of optical density after 60 min using the equation:

OD_0 _refers to the initial OD of the organism measured. Sixty min after OD_0 _was obtained, the cell suspension was centrifuged at 2000 rpm for 2 min. The OD of the supernatant was measured (OD_60_). Experiments were carried out in triplicate on two separate occasions [16].

*E. meningoseptica *isolates CH2B and NCTC 10016^T ^were examined for their ability to coaggregate with each other as well as with the following bacterial partner strains: *Aeromonas hydrophila, A. sobria, A. salmonicida, A. media, Acinetobacter *spp., *Enterococcus faecalis *ATCC 29212, *Escherichia coli *ATCC 25922, *Flavobacterium johnsoniae*-like spp. isolates YO12, YO19, YO51, YO60, YO64, *Listeria monocytogenes *NCTC 4885, *L. innocua *LMG 13568, *Micrococcus luteus*, *Pseudomonas aeruginosa, Salmonella enterica *serovar Arizonae and *Staphylococcus aureus *ATCC 25923 [11].

For coaggregation assays, bacteria were grown in 20 ml EAOB or TSB, harvested after 36 h, washed and re-suspended in sterile distilled H_2_O to an OD of 0.3 at a wavelength of 660 nm. The degree of coaggregation was determined by OD readings of paired isolate suspensions (500 μl of each isolate). The cell mixture was centrifuged at 2000 rpm for 2 min and the OD of the supernatant (600 μl) was measured at a wavelength of 660 nm [16]. The quantitative coaggregation rate of paired isolates was calculated using the equation:

where OD_Tot _value refers to the initial OD, taken immediately after the relevant strains were paired; and OD_S _refers to the OD of the supernatant, after the mixture was centrifuged after 60 min [16]. Experiments were carried out in triplicate on two separate occasions. Differences in coaggregation between *E. meningoseptica *CH2B and *E. meningoseptica *NCTC 10016^T ^were determined using one way repeated measures analysis of variance (ANOVA; SigmaStat V3.5). Differences were considered significant if *p *< 0.05.

### Reversal and inhibition of coaggregation

The effect of simple sugars, heat and protease treatment on isolate CH2B's ability to coaggregate with *L. innocua *LMG 13568 and *L. monocytogenes *NCTC 4885 was investigated.

The ability of simple sugars to reverse *E. meningoseptica *isolate CH2B coaggregation with *Listeria *spp. involved filter-sterilized solutions of lactose and galactose, respectively, being added to one of the coaggregating partners at final concentrations of 50 mM [17]. Mixtures were vortexed and tested for coaggregation using the coaggregation assay described above.

The ability of heat treatment to inhibit *E. meningoseptica *isolate CH2B coaggregation with *Listeria *spp. was conducted using the method of Kolenbrander *et al*. [18]. Cells were harvested from O/N EAOB/TSB cultures, washed three times and resuspended in de-ionized water. One of the coaggregating partners was then heated at 80°C for 30 min in a waterbath. Following heat treatment, the OD of each bacterial suspension was adjusted to 0.3 at a wavelength of 660 nm. Heat-treated and untreated cells were combined in reciprocal pairs and their capacity to coaggregate was assessed.

Protease sensitivity of the polymers mediating coaggregation of isolate CH2B with *Listeria *spp. isolates was tested using a method described by Rickard *et al*. [19]. Cells were harvested from O/N EAOB/TSB cultures and resuspended in de-ionized water to an OD of 0.3 at a wavelength of 660 nm. Proteinase K was added to the standardized cell suspensions to a final concentration of 2 mg/ml. Incubation at 37°C for 2 h was followed by centrifugation and washing of the pelleted cells three times in de-ionized water. Cells were resuspended and the OD adjusted to 0.3 at 660 nm. Protease-treated and untreated cells were combined and their capacity to coaggregate determined.

Differences in coaggregation between untreated *E. meningoseptica *CH2B and treated bacteria (*E. meningoseptica *CH2B, *L. innocua, and L. monocytogenes) *were determined by paired t-tests (SigmaStat V3.5). Differences were considered significant if *p *< 0.05.

### Induction of adherence

The standard microtiter plate adherence test [12] was modified to determine the ability of extracellular secretions of various aquaculture, food and/or human pathogens (*Aeromonas hydrophila*, *A. salmonicida*, *A. sobria*, *Chryseobacterium *spp. isolates CH8, CH15, CH23, CH25 and CH34, *E. meningoseptica *CH2B, *E*. *coli*, *Edwardsiella tarda*, *F*. *johnsoniae*-like isolate YO59, *L*. *innocua*, *L. monocytogenes*, *Myroides odoratus *MY1, *P. aeruginosa*, *S*. *enterica *serovar Arizonae, and *V. mimicus *VIB1) to induce enhanced adherence of *E. meningoseptica *CH2B.

Three-day old cultures of each of the above organisms were centrifuged at 2000 rpm for 10 min and supernatants were filter-sterilised using 0.2 μm filters, in order to obtain cell-free spent medium. *E. meningoseptica *CH2B cell pellets were washed and re-suspended in phosphate-buffered saline (PBS, pH 7.2) to a turbidity equivalent to a 0.5 M McFarland standard [11]. Ten μl of the standardized suspension was added to microtiter wells containing 100 μl TSB and 90 μl of the filtered supernatant. Controls included standardised isolate CH2B cell suspension added to TSB and respective filtered supernatants in TSB without isolate CH2B, in order to determine a change in adherence abilities and ensure that the change in adherence was due to induction, respectively. Microtiter plates were incubated at room temperature (21°C ± 2°C) for 48 h. An optical density (OD) reading of each well was obtained at 595 nm using an automated microtiter-plate reader (Microplate Reader model 680, BioRad Laboratories Inc., Hercules, California). Tests were done in triplicate on three separate occasions and the results averaged [12].

### Characterization of biofilm formation using flow cell systems

Biofilm formation by *E. menigoseptica *isolate CH2B was investigated using continuous culture once-through eight channel flow-cell system, while the mixed-species biofilm flow cell study involved *L*. *monocytogenes *strain NCTC 4885 together with isolate CH2B. The eight-channel perspex flow cell (channel size 30 × 4.5 × 3 mm), the glass cover-slip covering (no. 1 thickness, 75 mm by 50 mm), and attached silicone tubing (1 × 1.6 mm × 3 mm × 5 m tubing; The Silicone Tube, RSA) was assembled as described previously [11]. Silicone tubing was connected to a reservoir containing 2 l of EAOB/TSB and the flow cell was filled with EAOB/TSB, with a flow rate of 0.25 ml/min being maintained using a multi-channel peristaltic pump (Model 205S, Watson-Marlow, UK) located upstream of the flow cell. A one ml volume of EAOB overnight cultures of isolate CH2B was inoculated into each channel, below the clamps sealing silicone tubes upstream of each channel, using sterile syringes. One ml mixed pure culture inoculations, consisted of 0.5 ml combinations of *E. meningoseptica *isolate CH2B and *L. monocytogenes *NCTC 4885. Stagnant conditions were maintained for the first hour to allow attachment, prior to inoculated channels being exposed to flowing EAOB/TSB at a constant flow rate of 0.25 ml/min. Flow cell systems were kept at room temperature (21°C ± 2) throughout the experiments. Each flow cell channel was investigated by transmitted light using a Nikon Eclipse E400 (Nikon, Japan) microscope at 600-fold magnification and after 24 h and 48 h, respectively, to visualize bacterial attachment to a glass surface and biofilm development. Images were documented with a CHU high-performance charge-coupled camera device (model 4912-5010/000).

## Results

### Biofilm-forming ability of *E. meningoseptica*

*E. meningoseptica *isolates CH2B and NCTC 10016^T^, as well as *V. mimicus *VIB1 were screened for their adherence to polystyrene microtitre plate wells following 24 h incubation at room temperature (21°C ± 2°C) or 37°C, under static or dynamic conditions in nutrient-rich (TSB) or nutrient-poor (EAOB) media (Table 1).

**Table 1:** Biofilm formation by *Elizabethkingia meningoseptica *isolates CH2B and NCTC 10016^T ^following incubation at room temperature (~21°C) or 37°C, under static or dynamic conditions in nutrient-poor (EAOB) media or nutrient-rich (TSB), respectively

Parameters	**Biofilm formation (OD**_**595 nm **_**± SD)**^**a**^					
	*Elizabethkingia meningoseptica*				*Vibrio mimicus*	
		
	CH2B	**BF**^**b**^	**NCTC 10016**^**T**^	**BF**^**b**^	VIB1	**BF**^**b**^
**21°C EAOB dynamic**	0.23 ± 0.02	M	0.07 ± 0.01	N	1.34 ± 0.28	S
**21°C EAOB static**	0.31 ± 0.13	M	0.16 ± 0.01	W	1.01 ± 0.18	S
						
**21°C TSB dynamic**	0.44 ± 0.14	S	0.25 ± 0.01	M	0.11 ± 0.00	W
**21°C TSB static**	0.56 ± 0.06	S	0.26 ± 0.02	M	0.14 ± 0.07	W
						
**37°C EAOB dynamic**	0.38 ± 0.19	M	0.11 ± 0.07	W	0.30 ± 0.09	W
**37°C EAOB static**	0.46 ± 0.35	M	0.27 ± 0.06	M	0.37 ± 0.15	W
						
**37°C TSB dynamic**	0.84 ± 0.17	S	0.36 ± 0.02	S	0.34 ± 0.39	M
**37°C TSB static**	0.90 ± 0.07	S	0.42 ± 0.05	S	0.27 ± 0.34	M

^a ^Biofilm formation assay data is the mean of three independent experiments carried out in triplicate ± standard deviation following growth in minimal (EAOB) or rich (TSB) media at 21 or 37°C under dynamic or static conditions, respectively.

^b ^Biofilm formation (BF) was classified as non-adherent (N), weakly (W)-, moderately (M)- or strongly (S)-adherent using previously described criteria [12].

Isolate CH2B displayed moderate adherence in EAOB at both room temperature and 37°C, respectively, and became strongly adherent when exposed to TSB (Table 1). *E. meningoseptica *NCTC 10016^T ^was moderately adherently at room temperature and strongly adherent at 37°C in TSB, but weakly adherent in EAOB. In contrast, *V. mimicus *displayed strongest adherence in EAOB at room temperature. An increase in temperature to 37°C or alteration of the medium to TSB resulted in weak to moderate adherence for *V. mimicus *(Table 1). Given the small sample number, none of the physico-chemical parameter combinations resulted in statistically significant adherence.

### Bacterial hydrophobicity

Both *E. meningoseptica *isolate CH2B and *E. meningoseptica *NCTC 10016^T ^appeared to be strongly hydrophilic with BATH indices of 0.77% and 0.36%, respectively. Isolate CH2B was 'salted out' with a 4 M (NH_4_)_2_SO_4 _concentration, confirming its hydrophilicity.

### Autoaggregation and coaggregation indices

Isolate CH2B displayed an autoaggregation index of 37.4% (Table 2), while that of the type strain *E. meningoseptica *NCTC 10016^T ^was 33.1%. Coaggregation occurred to varying degrees between all of the 18 partner strains and *E. meningoseptica *isolates CH2B or NCTC 10016^T ^(Table 2), respectively. Isolate CH2B displayed coaggregation indices ranging from 2.5% with *E. meningoseptica *NCTC 10016^T ^to 82.2% with *S. aureus *ATCC 25923. Isolate CH2B had coaggregation indices >40% with 31.8% of the partner strains (Table 2). *E. meningoseptica *NCTC 10016^T ^displayed coaggregation indices ranging from 2.5% with *E. meningoseptica *CH2B to 75.1% with a *Micrococcus *spp. isolate. Strain NCTC 10016^T ^had coaggregation indices >40% with 42.1% of the partner strains (Table 2). Although, differences were observed in the coaggregation indices profiles of *E. meningoseptica *CH2B and *E. meningoseptica *NCTC 10016^T^, these were not statistically significant.

**Table 2:** Range of coaggregation indices obtained when partnering *Elizabethkingia meningoseptica *isolates CH2B and NCTC 10016^T ^with 19 diverse bacterial partner isolates

**Coaggregation partner strains**^**a**^	**Coaggregation indices (%)**^**b **^**with CH2B**	**Coaggregation indices (%)**^**b **^**with NCTC 10016**^**T**^
*Elizabethkingia meningoseptica *CH2B (37.4%)	37.4	2.5
*Elizabethkingia meningoseptica *NCTC 10016^T ^(33.12%)	2.5	33.1
*Acinetobacter *spp. (25.4%)	32.7	37.9
*Aeromonas salmonicida *(41.8%)	31.6	45.0
*Aeromonas hydrophila *(28.3%)	18.1	39.6
*Aeromonas media *(20.3%)	38.03	NT^c^
*Aeromonas sobria *(27.5%)	27.5	10.6
*Enterococcus faecalis *ATCC 29212 (45.0%)	44.3	6.9
*Escherichia coli *ATCC 25922 (20.8%)	39.5	32.3
*Flavobacterium johnsoniae*-like isolates		
YO12 (33.9%)	36.6	42.5
YO19 (16.1%)	28.9	13.0
YO51 (13.9%)	25.2	24.0
YO60 (27.5%)	18.0	38.2
YO64 (20.1%)	16.7	56.5
*Listeria innocua *LMG 13568 (56.2%)	77.4	47.7
*Listeria monocytogenes *NCTC 4885 (28.9%)	70.4	NT
*Micrococcus *spp. (51.1%)	37.2	75.1
*Pseudomonas aeruginosa *(24.3%)	44.1	40.5
*Salmonella enterica *serovar Arizonae (71.9%)	46.0	65.1
*Staphylococcus aureus *ATCC 25923 (76.1%)	82.2	68.9

^a ^For partner strains, autoaggregation indices were determined using assay described by Malik *et al*. [16] and are indicated within ().

^b ^Coaggregation indices represent the means of two independent replicate experiments as described by Malik *et al*. [16] and Basson *et al*. [11].

^c ^NT refers to not tested.

### Reversal and inhibition of autoaggregation and coaggregation

Since coaggregation indices of 70.4% and 77.4% were obtained between isolate CH2B and *L. monocytogenes *NCTC 4885 and *L. innocua *LMG 13568 (Table 2), respectively, they were selected for the reversal and inhibition of coaggregation assays following sugars, heat or proteinase K treatments.

Sugar reversal experiments with lactose or galactose of either partner increased both the autoaggregation and coaggregation indices (Table 3). Lactose treatment resulted in greater coaggregation of isolate CH2B with both treated and untreated *L. innocua *LMG 13568 compared with *L. monocytogenes *NCTC 4885, while this was reversed following galactose treatment, with greater coaggregation being observed with treated and untreated *L. monocytogenes *NCTC 4885.

**Table 3:** Reversal and inhibition of autoaggregation and coaggregation following sugar, heat or proteinase K treatment of *Elizabethkingia meningoseptica *CH2B, *L. innocua *LMG 13568, and/or *L. monocytogenes *NCTC 4885

Treatment	**Coaggregation indices (%)**^**a**^		
	
		Untreated	
	CH2B	*L. innocua*	*L. monocytogenes*
**Untreated**			
CH2B	37.4	77.3	70.4
**50 mM Lactose reversal **(*p *= 0.03)			
CH2B	68.7	96.0	89.6
*L. innocua *LMG 13568	94.5	-	-
*L. monocytogenes *NCTC 4885	87.8	-	-
**50 mM Galactose reversal **(*p *= 0.07)			
CH2B	56.4	86.2	95.7
*L. innocua *LMG 13568	85.0	-	-
*L. monocytogenes *NCTC 4885	95.8	-	-
**Heat inhibition (80°C for 30 min) **(*p *= 0.08)			
CH2B	20.9	33.5	12.9
*L. innocua *LMG 13568	93.8	-	-
*L. monocytogenes *NCTC 4885	97.7	-	-
**Proteinase K inhibition (2 mg/ml) **(*p *= 0.13)			
CH2B	25.8	38.2	5.8
*L. innocua *LMG 13568	80.4	-	-
*L. monocytogenes *NCTC 4885	94.6	-	-

^a ^Coaggregation indices represent the means of two independent replicate experiments as described by Malik *et al*. [16] and Basson *et al*. [11].

Heat treatment of isolate CH2B resulted in a decrease in autoaggregation (Table 3) and coaggregation, respectively. A greater decrease in coaggregation was observed with untreated *L. monocytogenes *NCTC 4885 than with untreated *L. innocua *LMG 13568. However, increased coaggregation was observed when the *Listeria *spp. partner strains were treated with heat (Table 3).

A similar trend was observed with proteinase K treatment of isolate CH2B, i.e., decreased autoaggregation of CH2B as well as coaggregation with the untreated partner strains. Proteinase K treatment of *Listeria *spp. isolates resulted in increased coaggregation between *L. monocytogenes *NCTC 4885 and isolate CH2B (Table 3). A greater reduction in the coaggregation indices were observed when heat- or protease-treated isolate CH2B cells were partnered with *L. monocytogenes *NCTC 4885 than with *L. innocua *LMG 13568.

### Cell-free supernatant induction of adherence

**Figure 1:**
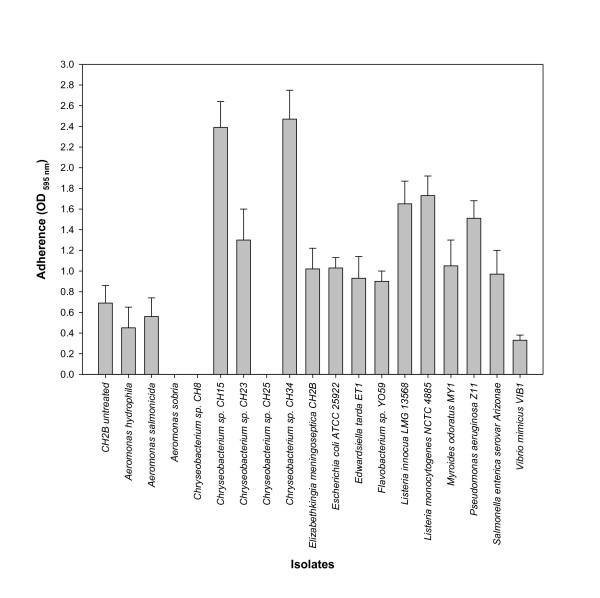
**Microtiter plate adherence of *E. meningoseptica *isolate CH2B, following exposure to cell-free spent medium supernatants from selected Gram-negative and Gram-positive bacteria, at room temperature (21°C ± 2°C) under static conditions in nutrient-rich (TSB) medium**. Bars represent means ± standard deviations for three independent replicate experiments.

Following exposure to cell-free supernatants from the three *Aeromonas *spp. isolates, *Chryseobacterium *spp. isolates CH8 and CH25 and *V. mimicus*, isolate CH2B's adherence decreased 0.48 - 1-fold (Figure 1). Increased adherence, ranging from 1.3 - 3.58-fold was observed with the remaining cell-free supernatants (Figure 1). Cell-free supernatants from *Chryseobacterium *spp. isolates CH15 and CH34, *P. aeruginosa, L. innocua*, and *L. monocytogenes *increased adhesion 2 - 4-fold. A 1.5-fold increase in adherence was observed following exposure of isolate CH2B to its own cell-free supernatant (Figure 1).

### Visualisation of biofilm formation using flow cells and microscopy

**Figure 2:**
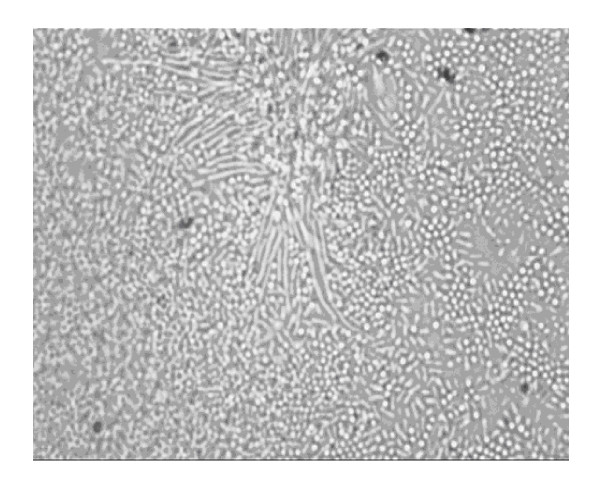
**Light microscope image depicting polar attachment (arrow) of *Elizabethkingia meningoseptica *isolate CH2B biofilm cells to glass slide surface following 48 h of incubation in nutrient-poor (EAOB) medium (× 1000 magnification)**.

**Figure 3:**
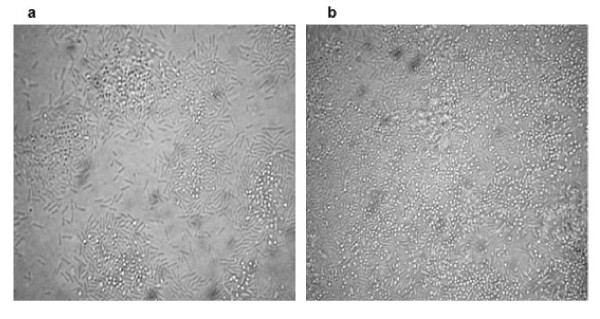
**Light microscope images depicting *Elizabethkingia meningoseptica *isolate CH2B cells associated with the glass slide surface as a) microcolonies after 24 h and b) complex biofilm formation following 48 h of incubation in nutrient-rich (TSB) medium, respectively (× 1000 magnification)**.

Adherence of isolate CH2B to glass coverslips was investigated by light microscopy, starting from the surface of the glass slide and scanning several planes interspersed by short distances in order to visualize biofilm architecture and microbial behavior throughout the depth of the individual flow chambers. By 24 h in nutrient-poor (EAOB) medium, isolate CH2B displayed initial widespread attachment to the glass coverslips and microcolonies were observed. After 48 h, majority of the cells were attached at a polar end (Figure 2), and cone-structures were observed with chains of cells reaching into the flowing medium. In nutrient-rich (TSB) medium flow cells, cells were attached along their length in microcolonies interspersed with polarly-attached cells (Figure 3a). Microcolonies merged by 48 h to form a thick, complex biofilm structure across entire channel surface (Figure 3b).

**Figure 4:**
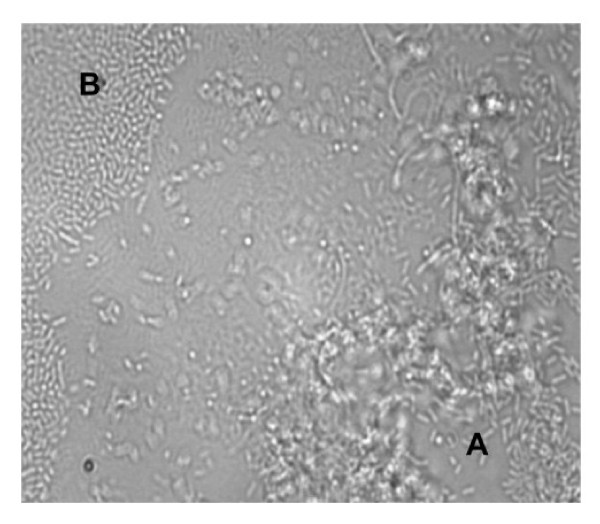
**Light microscope image depicting the distinctly separate adherence of *Elizabethkingia meningoseptica *isolate CH2B microcolonies (A) and *Listeria monocytogenes *NCTC 4885 cells (B) following 48 h in flow cells containing nutrient-poor (EAOB) medium (× 1000 magnification)**.

Distinction between bacterial strains in mixed-culture experiments was made visually by comparing images to that of pure culture, single-species flow cell experiments. Cells differed morphologically with *E. meningoseptica *CH2B cells being longer, thinner cells, and *Listeria *spp. shorter and thicker. When co-inoculated in nutrient-poor medium, both isolate CH2B and *L. monocytogenes *NCTC 4885 cells displayed delayed attachment to the glass surfaces, and attached cells were only observed 48 h following inoculation. Although both CH2B and *L. monocytogenes *NCTC 4885 cells were able to attach to the glass slides, distinct colonies were formed with no association between the different species (Figure 4). In nutrient-rich medium, cells of both species appeared to be scattered over the surface after 24 h, but by 48 h only a monolayer of isolate CH2B was observed covering the surface of the glass slide.

## 3. Discussion

*E. meningoseptica *has been identified in infection outbreak associated with municipal water reservoirs, potable water [7] and colonization of tap water in a neonatal intensive care unit [7]. Infections associated with *E. meningoseptica *have been associated with instrumentation contamination or the internal placement of indwelling medical devices [5,20]. Although its role in infection appears to be linked to biofilm formation and a worse outcome in patients [5], no studies have focused on investigating the factors involved in the adherence of *E. meningoseptica *to abiotic or biotic surfaces.

The presence of *E. meningoseptica *in various hospital environments involves optimal growth conditions including moist, cool environments or standing water at approximately 21°C [8]. Typically a shift to oligotrophic conditions triggers adhesion and biofilm formation [21], however, the converse was observed for *E. meningoseptica *CH2B. Unlike *V. mimicus *isolate VIB1, biofilm formation for *E. meningoseptica *was optimal in nutrient-rich TSB at both 21°C and 37°C, respectively. Lin *et al*. [5] also observed strong *E. meningoseptica *isolate-specific biofilm formation in the relatively nutrient-rich Luria-Bertani medium. A similar trend was observed for *Hafnia alvei*, where higher nutrient concentrations favoured biofilm formation [22]. *Myroides odoratus*, a related organism, by contrast, displayed strong adherence in both nutrient-rich and poor media at 21°C but was moderately adherent at 37°C in nutrient-rich medium [23]. Biofilm formation by avian faecal commensal *E. coli *strains was induced by both nutrient-rich and nutrient-poor media [24]. Even under nutrient-poor conditions at both 21 and 37°C, *E. meningoseptica *CH2B did not lose its ability to adhere, but displayed moderate biofilm-formation. Nutrient-poor conditions at lower temperatures and nutrient-rich medium at 37°C are conditions typically associated with environmental and clinical conditions, respectively. *E. meningoseptica *adherence occurred preferentially in nutrient-rich medium at both 21°C and 37°C, suggesting that nutrient limitation is not a cue in the switch to a sessile lifestyle for *E. meningoseptica*. Altering the hydrodynamic conditions appeared to affect the degree of biofilm formation more significantly in nutrient-rich medium and requires further investigation.

Whole cell hydrophobicity, autoaggregation, and coaggregation are important for colonisation and biofilm development in flowing environments [25]. Bacteria behave as hydrophobic particles due to their net negative surface charge and this surface hydrophobicity is usually associated with bacterial adhesiveness, varying from organism to organism, from strain to strain and is influenced by the growth medium, bacterial age and bacterial surface structures [26,27]. Although the general rule has been that adhesiveness increases and decreases with increasing and decreasing hydrophobicity, respectively [28], a number of studies have shown contradictory results where no relationship was found between the bacterial strain's surface hydrophobicity and the extent of initial binding to either a hydrophilic or hydrophobic substrate [14,29]*. Flavobacterium johnsoniae*-like and *F. psychrophilum *isolates from fish were hydrophilic by the BATH assay [11,27], as were adhesion-defective mutants of a *F. johnsoniae *strain displaying poor adherence [26]. Although *E. meningoseptica *CH2B appeared to be very hydrophilic by both the BATH and SAT assays, it displayed strong adherence.

The hydrophilic nature of the *E. meningoseptica *isolates might account for cells adhering preferentially along the entire surface of the glass slide rather than to the perspex surfaces in flow cells. Majority of the cells attached by their polar sides to glass in nutrient-poor medium, which could be an attempt to increase surface area for nutrient uptake in nutrient-limited environments, since horizontal attachment was observed in nutrient-rich medium.

According to Ofek and Doyle [20], capsule presence obscures cell hydrophobicity. Coagulase-negative *Staphylococcus *strains with capsules were more hydrophilic than non-encapsulated strains [30,31]. *E. meningoseptica *CH2B's hydrophilicity might be explained in part by the presence of a capsule layer (unpublished data). The capsule presence might also account for the autoaggregation index of 37%. Autoaggregation is a 'selfish' mechanism whereby a strain within the biofilm will express polymers to enhance the integration of genetically identical strains into biofilms [32], especially in high shear environments [25]. The high autoaggregation index could thus explain the aggregation of *E. meningoseptica *CH2B cells in the high shear inflow point of the flow-cell chambers.

Bacterial coaggregation is defined as cell-to-cell adherence of different bacterial species or strains [33]. Coaggregation plays an important role in the development of multi-species biofilms into integrated biological structures, by mediating the juxtaposition of species next to favourable partner species within taxonomically diverse biofilms [34]. The coaggregation profiles of isolate CH2B and strain NCTC 10016^T ^were not identical and this might be accounted for in part by the environmental and clinical isolation sources of the respective bacteria and diverse selection pressures potentially experienced in their diverse ecological niches. The strongest coaggregation partners with *Elizabethkingia *spp. isolate CH2B were not other Gram-negative bacteria commonly found in the aquatic environment, i.e., *Aeromonas *or *Flavobacterium *spp., but rather organisms important in food spoilage and/or intoxications, i.e., *S. aureus*, *L. innocua, L. monocytogenes, S. enterica, E. faecalis, and P. aeruginosa*. A similar trend was observed for *F. johnsoniae*-like isolates [11]. *M*. *luteus*, *B. natatoria*, *Fusobacterium *and *Prevotella *spp. have been identified as bridging organisms in biofilms due to their ability to coaggregate with diverse coaggregating partners [18,35,36]. In the present study, study isolate CH2B displayed high coaggregation indices with 12 of the 19 partner strains, and it is, therefore, not unlikely that it is a possible bridging organism in aquaculture environments.

Although both CH2B and *L. monocytogenes *NCTC 4885 attached to the glass slide in the mixed-species flow cell experiment, the high coaggregation index displayed by these two bacterial species was not apparent. The microcolonies of the two species appeared to be distinctly separated from one another on the glass surface. Based on induction experiment data, extracellular molecules in *Listeria *spp. growth medium supernatants, as well as that of *Chryseobacterium *sp. isolates CH15 and CH34, and *P. aeruginosa *increased the adherence of *E. meningoseptica *isolate CH2B to microtiter plate surfaces more than 2-fold. Quorum sensing signaling molecules within the cell-free supernatants could account for the increased adherence to polystyrene microtiter plates. This might also explain the high coaggregation indices observed with *Listeria *sp. isolates and the increased, albeit separate, adherence observed for both *L. monocytogenes *and *E. meningoseptica *CH2B in the mixed-species biofilm flow cell experiments. The 37.4% autoaggregation index, and the increased adherence observed for isolate CH2B following exposure to its own cell-free supernatant, suggests a potential role for quorum sensing in autoaggregation and biofilm formation.

Cell surface components or properties (flagella, pili, adhesin proteins, capsules, and surface charge) influence attachment and coaggregation. Flagella facilitate bacterial motility to specific attachment sites, while changes in cellular physiology affects surface membrane chemistry, surface proteins such as pili and adhesins, synthesis of polysaccharides, and cell aggregation, all of which influence adhesion [37]. Adhesive bacteria have developed various strategies to scaffold or present their adhesins. These include surface appendages and structures that bear adhesins, i.e., flagella, fimbriae, capsules, outer membranes, loosely attached peripheral components, etc. Adhesins may be proteins, polysaccharides, lipids, or teichoic acids [30]. The coaggregation interaction is a highly specific process mediated by the recognition of either complementary lectin (sugar-binding proteins)--carbohydrate molecules between the aggregating partners [33]; polysaccharides of capsule or LPS bind to lectins on host-cell surface; protein-protein; hydrophobic moieties of proteins on one cell binding with lipids on another cell; and/or lipid-lipid interactions. Receptors may contain carbohydrate or amino acid residues [30].

In order to investigate the type of adhesin structures present on the *E. meningoseptica *CH2B surface, inhibition of coaggregation assays were undertaken. Autoaggregation of CH2B cells was inhibited when untreated cells were paired with heat- or protease-treated cells. A similar trend was observed for *Acinetobacter calcoaceticus *[35]. Proteinase K treatment inhibited biofilm formation by non-typeable *Haemophilus influenzae*, as well as rapidly detached preformed biofilms [38]. Since both heat and protease treatments of *E. meningoseptica *CH2B resulted in decreased autoaggregation and coaggregation, heat- and protease-sensitive adhesins (lectins) appear to be localized on the *E. meningoseptica *cell surface. Attachment of heat- and protease-treated *E. meningoseptica *cells to untreated *L. innocua *and *L. monocytogenes *appears to involve different combinations of receptors, since variations were observed in the decreased coaggregation indices (Table 3). Heat- and protease-treatment of *Listeria *spp. cells resulted in increased coaggregation indices with untreated CH2B cells, indicating the presence of heat- and protease-stable listerial receptor molecules.

Sugar treatment did not produce a partial or complete inhibition of *E. meningoseptica *autoaggregation and coaggregation as observed for freshwater/aquatic bacteria [17,35,36] and sewage sludge bacteria [16]. Since the lectin-saccharide interactions are usually very specific, a wider variety of sugars might have to be assayed to yield a reversal of the coaggregation reactions. However, protein-carbohydrate interactions were not reversed by sugars [16]. The increased autoaggregation and coaggregation indices with both lactose and galactose were unexpected. This occurred when either isolate CH2B or the listerial cultures were treated with sugars. It might be speculated that the treatment sugars added to the capsular material enclosing isolate CH2B and intensified the adhesive effect and thus coaggregation. While capsule presence may mask potential adhesins such as fimbriae, it may stabilize the adhesion-receptor interaction. The capsule chemical composition, while primarily polysaccharide may also include protein adhesion molecules. Thus the capsule components may also be receptors for lectins on another bacterium [30]. Thus, in addition to conferring a hydrophilic nature to the cell, the capsule in *E. meningoseptica *CH2B might play an integral role in the strong adherence ability of this organism.

Factors affecting coaggregation include: adhesin and receptor density and distribution; hydrophobic character of receptor, adhesin or receptor nearest neighbours; medium composition and pH; and chelating agents [30]. Coaggregation among aquatic bacteria is mediated by lectin-saccharide interactions, and these aquatic strains often carry multiple adhesins or receptors or a combination of both, which is also a common feature of coaggregating oral bacteria [36]. Multiple adhesins may also be distributed around the *E. meningoseptica *cell surface allowing interactions with diverse microorganisms and colonization of diverse substrata. This would allow *E. meningoseptica *to compete successfully in a microorganism-rich environment.

The present study has shown that an *E. meningoseptica *isolate CH2B from tilapia possesses the ability to adhere to abiotic surfaces and form biofilms under various environmental conditions. Hydrodynamic flow in clinical or environmental niches may be more rapid than the rate of multiplication and unattached organisms will be eliminated, thus adhesion confers the important ability to colonise substrata [30]. *E. meningoseptica *CH2B was able to coaggregate with bacterial species important from a food and health perspective. Although *E. meningoseptica *are mostly described as opportunistic pathogens in both veterinary and human infections, the cause for concern arises from their association with pathogens and spoilage organisms causing great economic losses in the aquaculture and food industries and lethal device- or equipment-associated infections in immuno-compromised humans.

The ability of *Elizabethkingia *spp. to adhere to biotic and abiotic surfaces and the association with disease requires further study. Quantitative characterization and chemical analysis of the capsular material might provide valuable information regarding the capsule's role in the adherence abilities of *E. meningoseptica*. Furthermore, an investigation of specific cell-surface molecules mediating strong coaggregation abilities between *E. meningoseptica *and coaggregating partners may provide valuable information for anti-adhesion therapy which could be applied in aquaculture systems for the eradication of biofilms harbouring pathogenic organisms. The enhanced adherence of *E. meningoseptica *CH2B induced by cell-free supernatants points to the presence of a quorum sensing system, whose activity might be associated with autoaggregation, biofilm formation and/or the ability to colonise surfaces and initiate infection.

## Competing interests

The authors declare that they have no competing interests.

## Authors' contributions

AJ participated in designing the experiments, executing them, and performing data analysis. HYC conceived the study, participated in its design, data analysis and coordination and drafted the manuscript. All authors read and approved the final manuscript.
